# Giftedness and atypical sexual differentiation: enhanced perceptual functioning through estrogen deficiency instead of androgen excess

**DOI:** 10.3389/fendo.2024.1343759

**Published:** 2024-05-01

**Authors:** Kikue Sakaguchi, Shintaro Tawata

**Affiliations:** ^1^ Research Department, National Institution for Academic Degrees and Quality Enhancement of Higher Education (NIAD-QE), Kodaira-shi, Tokyo, Japan; ^2^ Graduate School of Human Sciences, Sophia University, Chiyoda-ku, Tokyo, Japan

**Keywords:** savant syndrome, gender identity, free-energy principle, hyper/hypoesthesia, autism spectrum condition, Klinefelter syndrome, estrogen deficiency syndrome

## Abstract

Syndromic autism spectrum conditions (ASC), such as Klinefelter syndrome, also manifest hypogonadism. Compared to the popular Extreme Male Brain theory, the Enhanced Perceptual Functioning model explains the connection between ASC, savant traits, and giftedness more seamlessly, and their co-emergence with atypical sexual differentiation. Overexcitability of primary sensory inputs generates a relative enhancement of local to global processing of stimuli, hindering the abstraction of communication signals, in contrast to the extraordinary local information processing skills in some individuals. Weaker inhibitory function through gamma-aminobutyric acid type A (GABA_A_) receptors and the atypicality of synapse formation lead to this difference, and the formation of unique neural circuits that process external information. Additionally, deficiency in monitoring inner sensory information leads to alexithymia (inability to distinguish one’s own emotions), which can be caused by hypoactivity of estrogen and oxytocin in the interoceptive neural circuits, comprising the anterior insular and cingulate gyri. These areas are also part of the Salience Network, which switches between the Central Executive Network for external tasks and the Default Mode Network for self-referential mind wandering. Exploring the possibility that estrogen deficiency since early development interrupts GABA shift, causing sensory processing atypicality, it helps to evaluate the co-occurrence of ASC with attention deficit hyperactivity disorder, dyslexia, and schizophrenia based on phenotypic and physiological bases. It also provides clues for understanding the common underpinnings of these neurodevelopmental disorders and gifted populations.

## 1 Introduction

Various neurodevelopmental atypicalities are overrepresented in males, and autism spectrum conditions (ASC), which have been the targets of primary attention, are approximately three times more prevalent in males than females (1) [however, some current arguments maintain equal prevalence of ASC across sexes (2)]. There are also global sex differences in professional interest and cognitive strength (3, 4). Classically, Geshwind et al. assumed that high prenatal androgen levels cause the suppression of “dominant” left cerebral cortex language field development, leading to learning disorders, left-handedness, and susceptibility to immune diseases and migraines (5, 6). In a more recent version, Baron-Cohen’s Extreme Male Brain (EMB) theory posits that systemizing-empathizing dimensions of cognitive preference explain ASC tendencies in parallel with male-female brain differences (7–9). There is a global sex difference in the 2^nd^ to 4^th^ digit ratio, and a smaller (< 0) ratio is regarded to reflect a stronger androgen effect *in utero*. The finger digit ratio is easy to measure and came to be the most popular putative biomarker of prenatal androgen effects, instead of handedness (10).

However, these premises are rebutted by many accounts that failed to find an association between various androgen measures and ASC symptoms (11), especially in ASC males (12). Brain differences between ASC versus typically developed (TD) in women somewhat overlap with male-female sex differences among TD, but they have little overlap with ASC-TD differences within men (13). The diagnosis of ASC does not imply a uniform tendency, which covers a multifaceted spectrum (14), questioning the plausibility of research strategies that investigate mechanisms in parallel with typical gender differences.

Therefore, we focused on multiple chromosomal/genetic syndromes associated with sex steroid deficiencies and the known risks of ASC. About 5–20% of ASC are considered syndromic (15), and many also show hypogonadism. Characteristically, 30–50% of individuals with Klinefelter syndrome (KS), one of the most prevalent syndromes with chromosomal aneuploidy, have ASC, in contrast to the prevalence rate of 1% in the general population (16). In other syndromes with hypogonadism, 11–80% of Prader-Willi syndromes (PWS) are associated with ASC (15). Fragile X and Down syndromes also show a high prevalence of ASC along with other neurodevelopmental disorder (NDD) symptoms and psychiatric status. Hundreds of genes related to developmental or psychiatric status overlap across different diagnostic categories (17, 18). The diagnostic criteria category is not as typological as previously believed. The viewpoint of capturing status as a spectrum covering diverse phenotypes has become predominant (14). When investigating the effect of sex steroids on cognitive function, it would be more promising to focus on syndromes with known physiological factors and common endophenotypes instead of being bound by the Diagnostic Statistical Manual criteria (19).

Another drawback of the EMB theory in explaining individual differences in strength and weakness across cognitive domains, in parallel with general sex differences, is that the prevalence of non-heterosexuals among the ASC population is nearly twice that of TD (20–22). At the same time, the prevalence of ASC in male and female transgender people is approximately 7–10 times than in the general population (23, 24). Furthermore, mediation analysis suggests that the non-heterosexuality of individuals with ASD is mediated by a higher incidence of gender dysphoria (20). Among KS individuals, some of whom experience hypogonadism *in utero*, and self-perceived gender nonconformity tends to co-emerge with NDDs (25). These pieces of evidence suggest a common underlying mechanism between NDD risk and atypical gender identity development.

**Figure 1:**
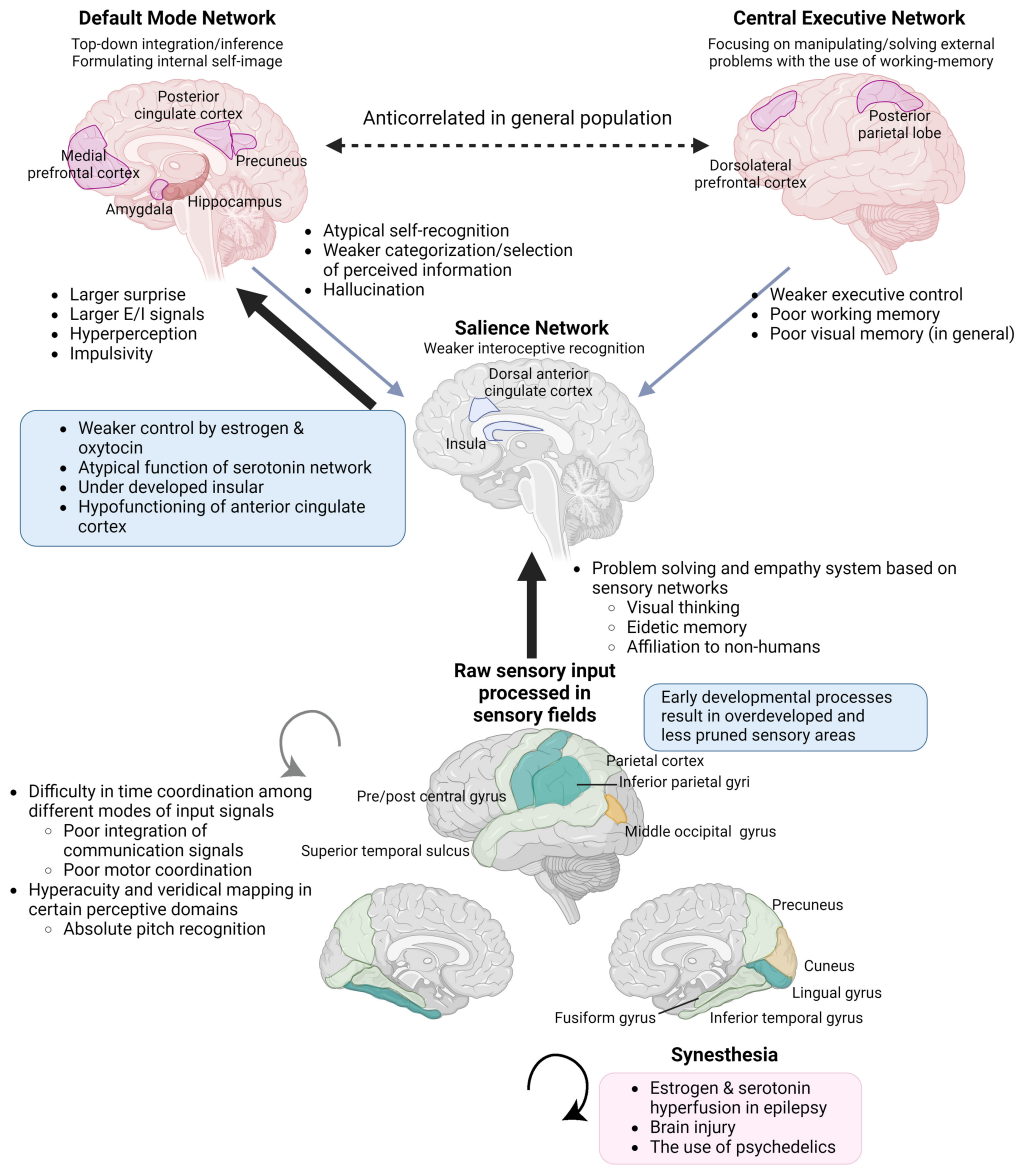
Integration of information processing theories and endocrinological characteristics underpinning endophenotypes of ASC, KS, and savant/gifted. The Enhanced Perceptual Functioning model and the neurodevelopmental theory based on the free energy principle, in combination with an understanding of the functions of brain networks and the interoceptive feedback system, can explain endophenotypes commonly observed across individuals with NDDs and savants/gifted individuals. The significance of each aspect varies across syndromes and subtypes within them. Brain images at the bottom. Yellow: the surface expanded areas in infants with high ASC risks. Light green: the thicker cortical areas in male to female transgenders. Green: the overdeveloped parts common to the two groups (Section 3.1.2). Individuals with KS also show similar overdevelopment patterns (Section 3.2.3). E/I, excitation/inhibition. (Created with BioRender.com).

In this paper, we have collected evidence that non-correlative biopharmacological studies support that *hypoestrogenism* is a more robust predictor of NDDs, and a cluster of outstanding abilities. The apparent correlation between hyperandrogenism and such cognitive traits can be explained as the result of negative feedback and a pseudo-relationship (26, 27) (see section 2.1.3). This paper connects evidence from atypical sexual development with *the neurodevelopmental theory* of ASC, which is based on *the free energy principle* by Karl Friston. Oxytocin plays an important role in early insular development by helping neural predictive signals synchronized with external and internal stimuli (28), leading to a sense of self-integration (29)(**Figure 1**). The insula is a central part of integrating external and internal sensations into self-image (28) and switches between the self-referential Default Mode and Central Executive Networks in the neural process of cognition (30). Steroids and oxytocin bidirectionally and synergistically affect early neural development and function in adults, and disruption of oxytocin activity has been reported in PWS, which shows hypogonadism and a strong tendency toward ASC (31, 32). In the rat brain, estrogen receptor beta (ERβ) is distributed over most cerebral cortex areas, mainly in layer V. Estrogen receptor beta is abundant in the primary motor and somatosensory fields, and has a lower density in the insula, followed by the cingulate cortex (33). The cingulate cortex works together with the insula and connects incoming sensation with emotional recognition and control. A recent hypothesis regarding schizophrenia suggested that insufficient estrogen and oxytocin levels are upstream etiologies. Moderate increases in these hormones ameliorate the positive and negative symptoms of schizophrenia by attenuating impairments in prepulse inhibition, resulting in the facilitation of emotion recognition and social interaction (34).

The brains of individuals with KS (16), ASC (35, 36), and schizophrenia (37) commonly show the under-development or function of the insula. Additionally, KS (16), ASC (38), and also male-to-female transgender individuals (39) share commonalities in the overdevelopment of primary sensory areas (**Figure 1**). Underdevelopment of the insula can lead to overfocusing on raw sensory input processed in sensory areas and an inability to abstract perceptive stimuli, resulting in shortcomings in emotional regulation, motor coordination, and the processing of communication signals, including language (28, 40, 41). This paradigm also helps to focus on the co-occurrence of endophenotypes across different diagnostic categories, such as ASC, attention-deficit hyperactivity disorder (ADHD) (42), schizophrenia, dyslexia, along with atypical self-integration, and mystical thinking, which might become the source of wild imagination and creative inspiration (43).

## 2 Androgyny hypothesis of ASC and underlying mechanism of cognitive phenotype

### 2.1 Estrogen deficiency instead of androgen excess hypothesis of ASC

#### 2.1.1 Syndromes with hypogonadism known as risk factors for ASC and other developmental uniqueness

The most notable drawback of the EMB is the high prevalence of ASC in several hypoandrogenic populations (44, 45). Klinefelter syndrome (XXY and its variations with >2 Xs) is the most prevalent sex chromosome aneuploidy (SCA), with a high end of prevalence rate of ≈1:500 in males (46). The lifetime diagnosis rate of KS is low, for example UK, 36% (47) and 23% (48); Australia, 50% (49); and Denmark, 25% (50), and approximately one-tenth of them are diagnosed prenatally (47). While typically known for tall stature, eunuchoid physique, learning difficulties, and impulsivity, the emergent phenotypes of KS are diverse. In a population who had been diagnosed irrespective of symptoms, the prevalence of gynecomastia and delay in school achievement did not differ from that in a control population (51). Common and robust characteristics include microorchidism, hypergonadotropic hypoandrogenism, and infertility (16). Hypoandrogenism is observed in > 75% of the diagnosed cases (16).

Among individuals diagnosed with KS, 30–50% are affected by ASC (16), and 63% are diagnosed with ADHD (52). The incidence of schizophrenia has been reported to be high; however, this observation was based on a small clinical sample (53). Psychosocial atypicality is generally considered to emerge together with hypogonadism features (54), whereas there have been case reports of ASC manifestation together with typical androgen production (55). A greater number of X-chromosomes leads to more pronounced physiological and cognitive characteristics (56, 57). In KS, ASC characteristics are mild, and restrictions of interest do not emerge (58). Verbal intelligence quotient (IQ) is typically in the low-normal range, whereas performance IQ measuring visuospatial ability is not impaired (59) and sometimes reaches a gifted range (>130) (60). Boys with KS show relative strength in arithmetic (59), although some report weakness in arithmetic problem solving. Executive function is affected by diminished cognitive flexibility and reduced working memory (53, 61). Testosterone supplementation improves language skills and concentration (53) (**Table 1**), but not visuospatial ability, which is generally considered to be promoted by androgens. Hypoandrogenism develops after puberty; however, speech and motor delays appear before that. Androgen secretion in the peripheral and central neural systems prenatally (165) and infantile mini-puberty (166) tends to be low, implying its influence on early developmental stages. Prenatal androgen production rate is diverse, as manifested in the micropenis manifestation in 10–25% of diagnosed cases (16). Hypogonadotropic hypogonadism cases are included in such a phenotype, and the percentage is as low as two in 160 KS individuals (167). Decrease in bone mineral density is observed in <40% individuals, indicating lower estrogen function (16).

**Table 1:** Sex steroid effects on cognitive domains with observed sex-differences.

Sense/cognitive domain	General gender bias	Responsible areas and receptors	Facilitating factor	Undermining factor
**Olfactory sensitivity/discrimination ability**	Female favor (62)	Medial amygdala (63) on significance evaluation AR (64) and ERβ (65)	Women in morning sickness (66) Ovulatory period in midcycle women (67) ASC (68, 69) Epileptic seizures (70)	Discrimination ability in women in morning sickness (71) ASC (72–76)
**Visual memory (copying complex figure)/visual attention**		Hippocampus AR and ERβ (77)	Androgen supply to FtM (78) E2 to androgen-deprived males (79) Oxandrolone to KS (80, 81)	ASC and other NDDs (82–85) TS, despite E2 supplementation (86) KS (87)
**Spatial memory** **(localization, remembering where thigs are located)**	Female favor	Hippocampus AR and ERβ (77)	DHT to hypogonadal men (88) Androgen in KS (80), might be partially mediated by ERβ (89) Oxandrolone to GDX male mice, partially through ERβ (89)	ERβ-KO in female mice (90) KS (80) Androgen deprived men (91)
**Mental rotation/problem solving (paper-folding)**	Male favor (92)	Frontal cortex for TD Visual cortex for ASC (93–95)	CAH women (96) Short AR CAG repeat lengths in TD men (97) Longer AR CAG repeat and larger 2D:4D in gifted boys (98) T levels intermediate of TD men and women (99–101) Heterosexual and gay men without childhood gender nonconformity (102) Low E2 in men (103)	CAH men (96, 104) High salivary T in TD men (97) High salivary E2 in women (105)
**Visual imagery**		Inferior parietal lobule esp. in non-dominant sphere (106, 107) Decreased activity of the Salience Network (insula and cingulate)	ASC (68), Savants (68, 108–113) Synesthesia (114, 115) Gastaut-Geschwind syndrome (112, 116, 117) Psilocybin on 5-HT_2A_ (118–120)	
**Spatial imagery**		“Where” pathway, dorsal posterior parietal cortex	Savants in ASC (121)	High salivary T in TD men (97)
**Object imagery: extracting patterns from visual noise**		“What” pathway, ventral inferior temporal cortex	Dyslexia (106, 122)	
**Language ability**	Female favor	Left occipital-temporal region Left frontal gyrus		ASC, KS (52, 123), dyslexia Diagnosed XYY (58)
**Verbal fluency**	Female favor		T to KS (53, 124) and hypogonadal men (88, 125)	ASC, KS, TS (86)
**Verbal memory**			TS, Methyltestosterone or Oxandrolone to TS (86) E2 to androgen-deprived males (79) T to hypogonadal men (88)	
**Social skills:** **in deficiency, ASC symptoms**	Female favor		Higher inhibin B in XYY (126) GABA_B_ agonist to FXS (127) T to KS (128)	PWS (15), FXS esp. PWP (127) ASC, KS and other SCAs, esp. with many X-chromosomes (56–58, 129) Diagnosed XYY (53, 58), TS (86) Down syndrome (130–132) PCOS in women and in the subjects’ mothers (133, 134) High androgen markers in women (135) Estrogen deficiency in the middle frontal gyrus (136) ESR1 SNPs in ASC (137) Low estriol and high maternal serum alpha-fetoprotein in pregnant mothers (138) Prenatal progestin and suppression of ERβ (139) High amniotic estrogen (11, 140) Prenatal dioxins and herbicides (141)
**Fine motor-skills/visual-motor integration**			Androgen in KS (53, 142)	ASC, KS, XXY (53), TS (86)
**Interoception**		Posterior insula: sensory integration Anterior insula and anterior cingulate gyrus: social self-recognition (143)	Oxytocin in human (29, 30, 144) E2 infusion in rat brain activates insula (145, 146) Generalized anxiety disorders (147)	ASC (28, 40, 41), KS (128)
**Self-awareness**		Cortical mediomedial structure in the DMN (148)		ASC (149, 150), schizophrenia
**Executive function**		Frontal lobe	T to KS, for concentration (53)	KS in cognitive flexibility (59) Hypogonadism in men (151) Manipulated X-chromosome numbers and GDX (77) ASC and other NDDs
**Working memory**				ASC, KS (152)
**Behavioral inhibition: opposite of impulsivity**		Prefrontal cortex (153)	E2 to androgen-deprived males (79) Increase with age (154)	KS, ASC, ADHD, and gifted (155) Androgen deprivation in TD men (156)
**Perseverance, risk-seeking towards rewards: Sensation-seeking**	Male favor (157)	Reward system (158)	Adolescent men (154)	
**Impulsive action** **(go/no-go task)**	Male favor (159)	Dopamine system	E2 in female rodents (160)	
**Impulsive choice** **(delay discounting)**	Female favor	Dopamine system Hippocampus	Orchiectomy (161) ADHD, addiction Hypogonadism, male prevalent NDDs Insufficient E2 in developmental stage (162)	Supraphysiological T administration (163) Women in follicular phase with E2 rise (164)

Gray shaded lows correspond to cognitive domains that are regarded as stronger in males.

2D:4D, 2^nd^ to 4^th^ digit ratio; 5-HT, 5-hydroxytryptamine (serotonin); ADHD, attention deficit hyperactivity disorder; AR, androgen receptor; ASC, autism spectrum condition; CAH, congenital adrenal hyperplasia; DHT, dihydrotestosterone; DMN, the Default Mode Network; E2, estradiol; ER, estrogen receptor; FtM, female-to-male transgender individuals experiencing significant body dysphoria (transsexuals); FXS, Fragile X syndrome; GABA, γ-aminobutyric acid; GDX, gonadectomized; KS, Klinefelter syndrome; NDD, neurodevelopmental disorder; PCOS, polycystic ovarian syndrome; PWP, Prader-Willi phenotype; PWS, Prader-Willi syndrome; SCA, sex chromosome aneuploidy; SNP, single-nucleotide polymorphism; T, testosterone; TD, typically developed; TS, Turner syndrome.

The XYY karyotype is rare, with approximately 1:1,000 males. They are characterized by tall stature, hypotonia, and cognitive problems; however, apparent symptoms are rare. Therefore, diagnosed individuals comprise a minority, with 0.7% (48), 5.9% (47), or at most 20% of XYY cases (168). Circulating testosterone levels are comparable to those in controls (48); however, infertility rate is higher than that in typical males (169, 170). There are sporadic reports of intersex states (testicular feminization) (171, 172) and transgender individuals (173) living as females. Among diagnosed individuals, cognitive and psychiatric profiles are similar to those of individuals with KS (53), but impairments in language and social responsiveness are more severe (58). To assess subtle differences in testicular function, particularly during prepuberty, inhibin B is a sensitive measure of testicular function, and lower inhibin B levels are associated with more autistic and problematic behaviors. Interestingly, pubertal rise in inhibin B is blunted, and prepubertal anti-Müllerian hormone levels are high, similar to those in KS. In XYY individuals, higher inhibin B or testosterone levels predict better cognitive, academic, and behavioral outcomes (126), contrary to the old expectation that XYY individuals are super-males and aggressive. Estradiol levels are low-normal (174, 175), and the osteoporosis rate is not high (48), suggesting that peripheral estrogen action is typical.

Individuals prenatally diagnosed with XYY show a higher-than-average IQ, in contrast to those who are postnatally diagnosed because of physiological/psychobehavioral problems. Postnatally diagnosed individuals with XYY show lower than average IQ and more ASC symptoms; however, physiological symptoms are comparable between the two groups. Although KS is characterized by shrunken testes, XYY males tend to exhibit macroorchidism, indicating a disrupted hypothalamic pituitary gonadal (HPG) axis (174).

Another genetic condition that connects hypogonadism and autistic cognitive predisposition is PWS. This is a genomic imprinting disease that lacks a paternally imprinted 15q11-q13 gene region due to mutations or maternal disomy. In contrast to the KS variations and XYY, but similar to XXXXY (57), the stature is short. Disruption of food intake regulation leads to obesity. Both males and females are since prenatally hypogonadal, adrenarche starts early, and the ASC prevalence rate is 11–80% (15). Personality problems are severe, with multiple learning difficulties and an IQ of 30–70 (176). Distinct from SCA is the inclination toward obsessive-compulsive disorders. Language delay is typical, as observed in the case of syndromic ASC, whereas special characteristics of PWS include the numbness to pain, vomiting stimuli, and temperature sensation. Additionally, individuals with PWS are very good at jigsaw puzzle (177). Therefore, locus 15q11-q13 has been considered to be responsible for savant syndrome (178), which includes relevant genes, such as gamma-aminobutyric acid type A receptor subunit gamma3 (*GABRG3*). Mutation of the gene affects the activity of the GABA_A_ receptor subunit, hindering inhibitory signaling in response to GABA. However, this finding was not replicated (179), implying that this region only explains a certain subtype of savant syndrome. Interestingly, individuals with PWS tend to be fond of caring for animals and babies (180), suggesting that they are not indifferent to their interactions with animate agents.

Fragile X syndrome (FXS) is a triplet repeat disease caused by the extension of CGG repeats in the fragile X messenger ribonucleoprotein 1 (*FMR1*) gene, which resides in the 5′ end noncoding region on the X chromosome. This is the most common cause of mental retardation in males, and the most common single genetic cause of ASC. Affected individuals show an elongated face, large ears, other physical malformations, and difficulty in swallowing food. The *FMR1* protein (FMRP) is one of the most abundant proteins in the brain and is crucial for synapse formation, regulating mRNA translation within dendrites. The mutation of *FMR1* targets GABA receptor subunits and downregulates GABA_A_ receptors. The protein is not synthesized when the number of CGG repeats exceeds 200, leading to obesity and behavioral problems. Anxiety, ADHD, and sensory hypersensitivity are frequent symptoms. The perseverance on topics is also frequent, sometimes meeting the criteria for obsessive-compulsive disorder. The abnormal enlargement of the testes begins at puberty. Significant to the current argument is the Prader-Willi phenotype (PWP) of FXS, which comprises 10% of all affected individuals; individuals with PWP have a syndrome similar to PWS, with heightened prevalence of ASC (54%) compared to 30% among males with FXS (127). The imprinting pattern of 15q11-q13 is normal; however, the cytoplasmic FMRP interacting protein 1 encoded by *CYFIP1* in 15q11-q13 colocalizes and works together with FMRP in dendrites to form neuronal structures, and the downregulation of *CYFIP1* results in this phenotype. In PWP, congenital hypogenitalism and hypogonadism emerge in 6 of 13 cases, with later development of macroorchidism in general (127). The full-scale IQ, including verbal and performance IQ ranges from to 36–49. In the molecular biological exploration of the common factors between FXS and ASC, the delay in synapse maturation, under-regulation of GABA_A_ receptors and subsequent imbalance of glutamine/GABA neural systems are considered primary causes (127).

Additionally, chromosome 21 trisomy (Down syndrome) shows comorbidities with ASC (1–42%) (130–132) and ADHD (34%) (130). Individuals with Down syndrome show characteristic facial features, low IQs, hypotonia, and poor motor coordination. In males with Down syndrome, approximately one-fourth show cryptorchism and 10% show hypospadias (181). Infertility is prevalent, and hypogonadism is also reported to be high; however, sex steroid levels are relatively normal in the postpubescent population. Approximately one-third of the individuals show elevated LH or FSH levels, indicating primary gonadal dysfunction (181, 182). Some individuals show savant skills, such as music (183), but considered to be rare. Symptomology related to ASC has eluded research attention in this syndrome.

In the general population, registry-based studies in Sweden demonstrated that individuals of both sexes with hypogonadotropic hypogonadism or delayed puberty showed a significant increase in the prevalence of ASC, ADHD, and intellectual disabilities compared to matched controls (184). The impact of hypogonadism remained significant even after excluding individuals with chromosomal abnormalities from the analyses. Kallman syndrome is a genetic disorder that manifests with outcomes associated with congenital hypogonadism. Affected individuals show hypogonadotropic hypogonadism, anosmia, an elongated face, large ears, and an increased risk of osteoporosis, hearing loss, mental handicap, and schizophrenia (151). Gonadotropin-releasing hormone-1 (GnRH-1) neurons originate from the nasal placode, and atypical development at this earliest neural development stage leads to disorganization of HPG axis. Subsequently, several ASC risk genes require the facilitation by androgen or estrogen to fulfill their typical neurodevelopmental functions, as discussed in the next section. Additionally, testosterone (185) and estrogen (see Section 2.1.3) directly modulate GABA_A_ receptor function, and their deficiency leads to destabilized excitation in the limbic system.

Androgen deficiency affects hypogonadal populations mainly on impairing communication abilities. Weaker activation of the dopaminergic reward circuit (see section 3.3) and the amygdala (see section 2.1.3) likely induces interpersonal anxiety and weaker interpersonal motivation. Impairment in executive control of behavior through weaker control of the prefrontal cortex (see section 3.3.2) and in spatial memory through hippocampal underdevelopment (3.2.1) are also possible outcomes. The effect of androgen is partly through AR and partly through ERβ in non-aromatized or after aromatized form.

#### 2.1.2 Cognitive characteristics explaining ASC-savant traits and major possible target gene expressions triggered by sex-steroid deficiency

The free energy principle is a broad theory in computational neuroscience that explains the formation of neural circuits and cognitive processes as the minimization of gaps between the expectation of signals and reception of input. As a comprehensive theory similar to Hebb’s rule, the free energy principle tries to explain how the information from stimuli is joined with the inner process representing phenomena in neural systems, and the closing gaps updates neural wiring. The principle was originally formulated to explain NDD phenomena, such as ASC and schizophrenia, and explains the characteristics of these two apparently different symptoms in the same model with different parameters. This principle has been demonstrated to be applicable to the decision-making process through the modulation of dopamine receptors (186), and to the learning process through cholinergic neuromodulation (187).

**Figure 2:**
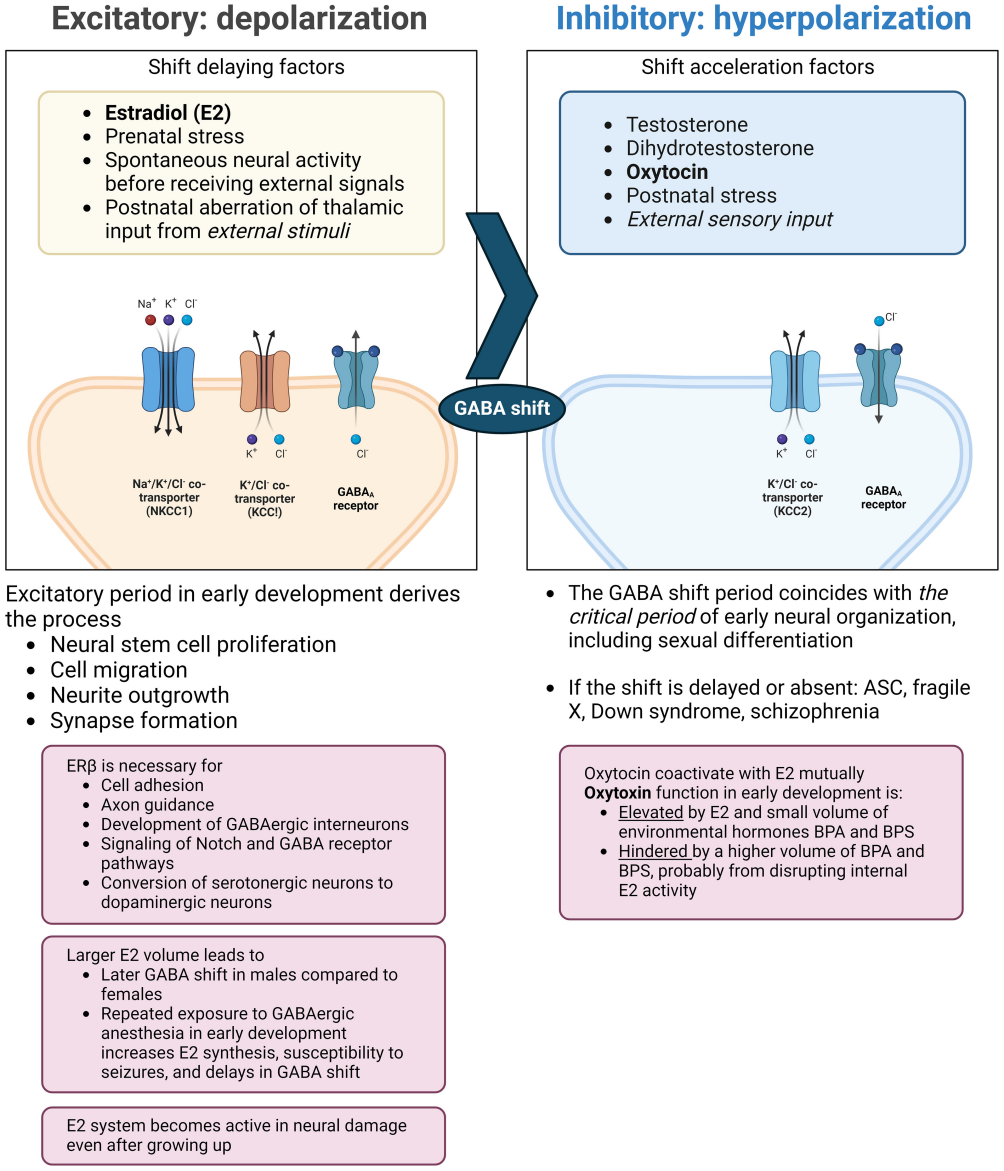
Endocrinological environment affecting the timing of GABA shift. Perturbation of ERβ activity hinders neural and glial cell development and adhesion. This also interrupts the function of oxytocin (right pane) and delays the timing of the GABA shift. The extended immature phase of the brain corresponds to weaker GABA inhibition and the skew to the serotonergic system compared to the dopaminergic system. Also, excessive estradiol doses by endogenous/exogenous causes a delay in GABA shift and can induce epilepsy, with possible subsequent distortion of steroidogenesis and functional pathways. ASC: autism spectrum condition; BPA: bisphenol A; BPS: bisphenol S; ER: estrogen receptor; GABA: γ-aminobutyric acid; KCC: K^+^-Cl^-^ cotransporter; NKCC: sodium-potassium-chloride cotransporter. (Created with BioRender.com).

The neurodevelopmental hypothesis derived from the principle posits that a major causal factor of ASC symptoms is the disruption of *GABA shift* in early development (41) (**Figure 2**). The oxytocin-induced GABA shift changes GABAergic neuronal function from excitatory (depolarizing) to inhibitory (hyperpolarizing). Estradiol, especially its work on ERβ, is essential for organizing neural distribution in the developing brain (188, 189). Excessive estradiol delays GABA shift (190–192), and steroidogenesis dysfunction also delays this shift. For example, high concentrations of environmental disruptors, such as bisphenol A or bisphenol S hinder oxytocin function and GABA shift, likely by disrupting endogenous estradiol function (193). The delay or elimination of the shift leaves the brain in an immature state, leading to inflation of excitation/inhibition (E/I) ratio and a delay in closing *the critical period* postnatally, that is supposed to prune excess synapses, affecting neuronal plasticity (194). This leads to microstructural atypicality and hypoconnectivity of neural circuits around the amygdala, insula, and cingulate cortex, as well as psycho-behavioral atypicality. The GABA shift is an event that changes the brain from an organizational neural distribution phase to a synapse pruning phase in accordance with external sensory input (195).

In contrast, the major psychological explanations of the cognitive characteristics of ASC, covering communication deficiency and higher performance in certain visual tasks, have been: 1) the Weak Central Coherence (196, 197); 2) the Enhanced Perceptual Functioning (EPF) (198, 199); 3) systemizing the brain through the EMB (7); and in a classic form, 4) the Geschwind–Galaburda hypothesis, which attributes the cause to the imbalance of hemispheric development (5, 6). The Geschwind–Galaburda hypothesis explains that higher prenatal androgen levels cause a delay in the development of left cerebral hemisphere, as manifested by an increase in non-right-handedness and atypical brain torque in individuals with ASC (200). The neurodevelopmental hypothesis indicates the order of development of these phenomena: the difficulty in closing gaps between internal and external perceptive stimuli and the expectation of received signals leads to non-optimal attenuation of perception, as manifested in hyper/hypoesthesia 2), and difficulty in summarizing perceived information 1), resulting in skewed strength/weakness in cognitive domains 3) and the skewed development of corresponding brain areas 4). Steroid deficiencies in early development and adulthood are possible causes of failure to attenuate neural signals. This could be manifested by overexcitation over primary perceptual input (201), overdevelopment of the visual cortex and primary sensory areas (38), glial cell atypicality perturbing synapses and neural circuit maturation (202–204). Delays in synapse maturation, reduced synapse pruning, and atypical neural networks of glutamine and GABA (205, 206) are hallmarks of ASC.

In higher-level cognitive processing, insular malformations are key to understanding the hindrance to integrating higher-order ego recognition and naming emotional experiences of self and others (36). The insula integrates multimodal sensations from the outside world and inner body into self-awareness. The posterior insula is an integral part of passing *interoception* to the brain, and it comprises visceral feelings and feedback from the autonomous nervous system. Disconnection from the interoception network leads to *alexithymia* (207). The inability to anticipate one’s own bodily responses leads to easy panicking and shyness (128). Although ERs are not densely distributed, estrogen infusion into the insula of the rat brain excites neurons by suppressing GABA release in this area and activates descending sympathoexcitatory pathways (145, 146). On the other hand, higher oxytocin concentrations correspond to stronger feelings of body ownership (29), sensitivity to the social environment (144), and emotional empathy in humans, possibly by strengthening the interoceptive network and attenuating physiological reactions to negative stimuli. Testosterone supplementation in individuals with KS improves communication and interpersonal skills, possibly by smoothing the expectation of perceptual input and helping integrate socially relevant information, thereby reducing anxiety about interacting with others (128, 208).

Major ASC-susceptible genes are modulated by estrogen and androgen, and steroid deficiencies can cause diverse cognitive symptoms. Forkhead box protein P2 gene (*FOXP2*), originally discovered in a family line with language developmental disorders, and its paralog, *FOXP1*, the fifth major risk factor for ASC and a common risk factor for other NDDs (209), are among them. *FOXP2* is expressed in earliest-born cortical neurons in the subplate, and the protein binds to DNA to facilitate neural development and cell-type differentiation (210). Non-aromatizable androgens are necessary for normal expression and function of Foxp1 and Foxp2 in rats (211). *Foxp2* is expressed in the sensory and motor-related cortices, cerebellum, and medial amygdala. Knockdown of *Foxp2* in mice compromises social behavior processed in the medial amygdala, in dopamine-dependent manner (63). In female zebrafish, *Foxp2* deficiency leads to disruption of the HPG axis (212), and *FOXP2* is overexpressed in human prostate cancer cells (213). These results suggest that FOXP2 functions in the feedback on estrogen and androgen pathways.

Additionally, SH3 and multiple ankyrin repeat domains 3 (*SHANK3*) is another major gene factor against ASC, regulating neuronal and synaptic excitability (214). In line with *SHANK1* and *SHANK2*, it functions on *the N*-methyl-D-aspartate (NMDA) and glutamate receptors, and is involved in dendritic spine maturation (215). SHANK3 is crucial for fixing and guiding the actin cytoskeleton of neurons before synaptic transmission; knockdown of *SHANK3* reduces neuronal soma size, growth cone area, neurite length, and branch numbers (216). In macaque monkeys with the ASC-type mutant *SHANK3*, behavioral outcomes include sleep disturbances, motor deficits, repetitive behaviors, and social and learning impairments. Neuronal networks were also altered into hypo-connectivity in the Default Mode Network (DMN: see section 2.2.1) and local hyper-connectivity in areas including the somatosensory and posterior cingulate cortices (217). In human cell culture, dihydrotestosterone (DHT) increased the expression of *SHANK* by 35% and estradiol by 15%, indicating that both AR, ERα, and ERβ contribute to regulation (218). In contrast, Srancikova et al. found that testosterone downregulates SHANK1 and SHANK3, and that gene expression was lower in the hippocampus of male than of female rats (219).

In rodents, estrogen regulates sexual characteristics, puberty, and neurobiological reproductive systems through ERα. Contrary, estrogen modulates non-reproductive systems, such as anxiety, locomotion, fear, memory, and learning, through ERβ. If the action of estrogen on these neural systems during developmentally critical stages is insufficient, the typical development of neurons, synapses, and glial cells is hindered (220). The action of ERβ is not necessarily estrogen-dependent (188); the androgen metabolite androstanediol is also suggested to promote oxytocin function through ERβ (221). In human brain, areas with especially abundant ERβ are somatosensory cortex, hippocampus, thalamus, and cerebellum (222). Insufficient estrogen action is a possible causal factor for various psychiatric and NDDs that emerge with different sex ratios after puberty. In women, psychiatric syndromes, including schizophrenia, ASC, ADHD, and general anxiety disorders are exacerbated when estrogen levels are low (222).

#### 2.1.3 Estrogen deficiency sometimes emerges with an increase in androgen markers in males

Differentially expressed gene analysis across different phenotypes of ASC revealed that both androgen and estrogen signaling pathways are related to these conditions. Androgen signaling is associated with the emergence of a savant tendency (223, 224). Among ASC related genes, scavenger receptor class B type 1 (*SCARB1*) which codes a membrane protein regulating cholesterol usage in cells is also involved in sexual differentiation, and 5α-reductase type 1 (*SRD5A1*) gene controls cellular cholesterol intake and testosterone metabolism (224). However, these two genes have relatively small explanatory power among ASC-related genes (209). An apparent increase in steroid biomarkers may be secondary to a deficiency in sex steroid action during critical developmental periods and the subsequent disruption of steroidogenesis. Additionally, steroids functions not only trigger genetic expression cascades through classical actions on nuclear steroid receptors. Instead, many estrogenic neuromodulating mechanisms seem to depend on receptors on the membrane, which directly and rapidly modulate the strength of GABA or glutamate signal transmission in neurons (225, 226), and the activities of glial cells (98, 227–229), which guide neural extension, synapse modulation, and information transmission.

The G protein-coupled estrogen receptor (GPER) is a major estrogen membrane receptor expressed in primate LH-releasing hormone neurons in the olfactory placode and hypothalamus, modulating the HPG axis (230). Male HPG axis negative feedback loop is mainly controlled by estrogen instead of non-aromatized androgen in pituitary, suggesting the insufficient estrogen action behind the gonadotropin increase (231). GPER plays an important role in the control of spermatogenesis in the testes (231). Individuals with KS typically show hypergonadotropic hypogonadism, and their testicular tissue shows a 12-fold increase in GPER and a decrease in ERβ mRNA expression compared to control males, indicating insufficient estrogen control for testicular function in this population (232). GPER is expressed in both the central nervous system and peripheral tissues, including the cardiovascular system. Among children with ASC, serum GPER levels decreased with an increase in symptom severity. However, serum estradiol levels did not correlate with GPER levels (233).

During the developmental phases in men, excess androgen levels trigger negative feedback or are buffered within the high-normal range, which does not necessarily deliver unique developmental characteristics, as expected in women. For example, fetuses with congenital androgen hyperplasia (CAH) are exposed to androgens at supranormal concentrations in the adrenal glands because of a lack of metabolic pathways. This causes physiological and psychological masculinization (234, 235) only in females, but *does not increase* the odds of ASC, according to a recent meta-analysis (133).

When mothers suffer from polycystic ovary syndrome (PCOS) and androgen production is elevated, the conceived babies’ risk of developing ASC increases (134). Animal models suggest that in girls, the elevation of prenatal exogenous androgen levels is likely to induce postnatal upregulation of androgen production and activity, whereas in males, this induces a decrease in luteinizing hormone (LH) and underdevelopment of the testes (236). Estrogen concentrations in affected mothers and offspring are not consistently altered, but aromatase activity *decreases* in the placenta of mothers (237). In mice, the offspring of androgen-exposed mothers show downregulation of AR or ERα in a sex-dependent manner in the hypothalamus, hippocampus, and amygdala, showing anxiety symptoms. The expression of serotonergic and GABAergic genes tends to increase in a sex-dependent manner (238). A survey of gifted boys, who would have a common physiological background with savants reported that they had smaller 2D:4D finger ratios, suggesting the influence of higher androgens in the prenatal environment. However, salivary testosterone levels were significantly lower, and performance on reading in the eye test was poorer (239). This case might correspond with the case of offsprings who have been exposed to exogenous androgen *in utero*, while in male neonates, the association between amniotic testosterone levels and the 2D:4D ratio is questioned (240). Additionally, an initial report of smaller 2D:4D ratios in individuals with autism (241) was not replicated (12), or was limited to syndromic cases among men (242).

In an attempt to distinguish adults with Asperger’s syndrome from those typically developed with 24 serum biomarkers, only four markers were common between men and women. An increase in LH and free testosterone levels was observed only in women; among men, the main characteristics were higher levels of cytokines and other inflammatory measures (135). Geschwind et al. pointed that ASC and gifted are often suffering from autoimmune diseases (5). Autoimmune diseases are more prevalent in females compared to males in the general population. However, long-term aromatase deficiency induces autoimmune diseases in mice (243).

Congenital estrogen deficiency results from the deletion of the aromatase gene cytochrome P450 family 19 subfamily A member 1 (*CYP19A1*) or abnormal function of ERs (estrogen resistance). The PWP of FXS, who are at high risk of ASC (127), shows aromatase deficiency, with eunuchoid proportions, early onset metabolic syndrome, and oligozoospermia, similar to KS, in combination with cryptorchidism or *macroorchidism* (see section 2.1.1). Among various endocrinological diseases with the depression of estradiol levels, aromatase deficiency causes a large drop in estradiol levels; testosterone levels are low in some cases, and high in others (26). Mothers with fetuses affected by aromatase deficiency show virilization during the third trimester. Congenital estrogen deficiency in women leads to virilization of the genitalia, decreased estrogen levels, and increased androgen levels (27). In rats, prenatal exposure to synthetic progesterone in the form of oral contraceptives results in ERβ suppression in the amygdala and ASC-like behavior (244). Estrogen deficiency leads to insulin resistance, which hinders synaptic plasticity and dopaminergic function in the ventral striatum, thereby inducing anxiety and depression. Hypoactivation of the mesocorticolimbic and nigrostriatal dopamine pathways has been suggested to correspond to low social interaction motivation and stereotyped behavior (245).

Deficiencies in ERβ, CYP19A1, and ER coactivators in the middle frontal gyrus can be a direct cause of ASC (136). Acid-related orphan receptor alpha (RORA) is a transcription factor that induces aromatase expression through a feedback loop at sex steroid concentrations (246). In ASC population, RORA and aromatase expression are greatly decreased (247, 248). In a survey of the Japanese population, single nucleotide polymorphisms (SNPs) of ESR 1/2 were found to be related to the severity of symptoms in ASC. Human ER genes, ESR1 and ESR2, encode receptors that are homologues of ERα and ER β respectively, and ESR1 was concerned with the impairment of social interactions, and ESR2 with emotional regulation. However, ESR1/2 SNPs did not predict the severity of social communication problems, stereotypies, or sensory abnormalities (137). Among male and female transgender populations, which tend to co-emerge with ASC, many gene variants correspond to estrogen signaling pathways in sexually dimorphic brain areas, but none to androgen pathways (249).

In the prenatal environment, low concentrations of unconjugated estriol (uE3) and high concentrations of maternal serum alpha-fetoprotein (MSAFP) in the maternal serum increased the odds ratio of ASC prevalence in offspring (138). A low uE3 concentration is an indication of insufficient production of adrenal steroid (dehydroepiandrosterone and others) in infants, and MSAFP suppresses estrogen activity. Additionally, prenatal exposure to progestin, which is prescribed to prevent threatened miscarriage, has been reported to suppress the expression of ERβ in the fetal brain, thereby increasing the risk of ASC in rat experiments and epidemiological studies (139).

The above evidence suggests that one of the main factors that induce ASC is the depression of estrogen action; if the potential for steroidogenesis in the fetus is intact, the androgen production rate would be increased to compensate for estrogen deficiency. In another case, an increase in exogenous androgen circulation downregulates endogenous androgen genesis in the fetus, that will cause estrogen deficiency subsequently; however, using a Danish cohort sample, Baron-Cohen et al. showed an increase in various estrogen and progesterone concentrations in amniocentesis fluids of boys with ASC (11, 140).

In addition to syndromic ASCs that lack steroid hormones during early development, there are known cases of externally caused shortages of sex steroids that induce ASC. The responsible environmental disruptors are dioxins and herbicides (141). An analysis of umbilical codes suggests that exposure to high levels of dioxins suppresses androgen production in male fetuses (250). Bisphenol A (BPA) is a blocker of AR (251) and ER, affecting the expression of multiple ASC related genes. Bisphenol A exposure in pregnant rats increased neurite length and the number of neurite branches in offspring of both sexes, while an increase in neuronal cell death, the impairment of neuronal development in the hippocampus and learning ability were observed only in male offspring (252). The prefrontal cortex of adult Long-Evans rats prenatally exposed to high concentrations of BPA showed an increase in the number of neurons and glia in layers 5/6, but only in males (253). In contrast, an epidemiological test using a public human cohort demonstrated that the effect of BPA on ASC susceptibility was more apparent in girls (254).

Studies have reported the disruption of steroidogenesis in individuals with ASC, particularly alterations in metabolic pathways before steroids are converted into sex hormones (255). Along with estrogen, neurosteroids such as the progesterone metabolite allopregnanolone, are crucial for neural cell proliferation, migration, myelination, synapse formation, and modulation of GABA_A_ receptors in the cerebral cortex, thalamus, and hippocampus (256, 257). This function persists from the earliest stages of brain development to maturity. Androgens show similar functions or work in consort with estrogen, but their importance in early neural development and cognitive modulation seems to be secondary, as direct pharmacological evidence of the effects of developmental androgen alteration is limited. Such reasoning resolves the discrepancy, especially in affected males, where researchers have largely failed to find an increase in androgen markers prenatally or postnatally (133, 255).

One epidemiological support for the early idea that an abnormal increase in steroidogenesis causes ASC comes from the fact that peripubertal (15–19 years) individuals with ASC have a higher incidence of genital/ovarian cancer. Mothers of individuals with ASC also frequently experience sex hormone-responsive cancers (258). The increased vulnerability to disease, likely derived from the abundance of sex steroids, is more evident in female ASC than that in male ASC cases. Some ASC-related genes, such as *FOXP1* and copy number variation, are known to suppress tumors or cancer if they are intact, requiring further examination to explore how such a genetic background interacts with steroid excess contributing to cancer risk.

### 2.2 Extreme success in “masculine” artistic-academic fields inversely correlate with masculinity indices among men

#### 2.2.1 Connection between developmental disorders, savant/gifted, and atypical sexual differentiation

Approximately 50% of individuals with savant ability are from the ASC population (259), and the emerging ratio of sex differences is approximately three men to one woman (260). Savant ability is independent of total IQ scores. In the general population, *prodigious savants* who show distinguished talents that are difficult to interpret with common sense, are very rare, with only approximately 50 individuals worldwide. If the definition is expanded to *talented savants* who show some distinguished abilities relative to other domains of talent of their own, the prevalence among the ASC population is estimated to be from 28.5% (260) to 42% (261) or nearly 50% (262). In the early stages of academic notice, savant art was considered as an emotionless repetition of the gross amount of memory without original creativity. This notion was later corrected; savant art has its own originality, and is considered a model of the root of creativity (262, 263).

In contrast, gifted individuals are defined as “those who show exquisite talents in one or several fields (logical thinking, learning ability, etc.), or those who show abilities of top 10 scores of a population (264).” The greater the extraordinary ability, the greater tends to be the developmental unevenness across cognitive domains and social maladaptation. The behavioral output of gifted individuals is often difficult to distinguish from or co-exists with ADHD, obsessive-compulsive disorder, ASC, schizophrenia, and avoidant personality disorders (155). Whether an individual is judged as savant or gifted is largely influenced by the academic background of the report.

Using the Bayesian explanation of the free energy principle, hypopriors (having fewer internal models prior to processing stimuli) can explain hypersensitivity to sensory input, immunity to being distracted by contextual information (better at copying impossible figures), and incompetency in communication dependent on summarized inference (265) (**Figure 1**). Therefore, the EPF model in combination with the free energy principle does not require supposing a lack of empathic motivation in the first place, but suggests that the characteristics of perceptual processing are the basis of both unevenness in the strength of cognitive domains and communication inaptness.

The main premise of the EBM theory is that higher androgen action on the prenatal brain increases ASC traits *between and within* sexes. However, ASC traits are more observable among hypoandrogenism conditions, contrary to expectations, except for females with PCOS and offspring of mothers with PCOS (133). The effects of maternal PCOS on offspring are under debate, with complications from epigenetic effects, genetic inheritance, insulin resistance (266, 267), and the effects of metformin administration on mothers (268). Additionally, Baron-Cohen et al. admit that MRI voxel-based morphological ASC-TD brain differences within sex are not parallel between sexes, and the difference within men is especially discordant with TD sex differences (13). They also reported in adults, blood serum biomarkers, such as high LH and free androgen index could distinguish between women with Asperger’s syndrome and controls; however, the same marker set did not have discrimination power for men (135).

The DMN is the functional connection of brain areas involved in self-referential mind wandering, which becomes active when a person is not focused on performing tasks. Researchers have become increasingly interested in the DMN because of its ability to discriminate atypical cognitive statuses. Baron-Cohen et al. compared the connectivity strength of the DMN between sexes in TD and within sexes across ASC and TD groups. They reported that TD males and individuals with ASD had weaker connectivity in the DMN (269). In contrast, in an analysis of whole-brain connectivity without presupposed theories, the connectivity between social brain areas (fusiform gyrus, superior temporal sulcus, inferior parietal cortex, insula, and posterior cingulate cortex) and other areas was stronger in female ASC (male-like), but weaker in male ASC (female-like) (270). They argued that this result supports the atypical sexual differentiation theory of ASC genesis instead of the EMB theory.

Do gifted individuals tend to be hypermasculine or gender neutral? Several studies support the latter tendency. The fields of interest and strengths of gifted individuals tended to be gender-neutral instead of following stereotyped gender categories (155, 271). Their gender identities also tend to be neutral (272). Another survey showed that neutral gender identity was more evident among gifted women than in men (273). Additionally, more students in gifted classes recognized themselves as sexual minorities than their non-gifted counterparts (274).

## 3 Expectation mismatch on perceptual input will lead to uneven development of perceptual processing and difficulty in information integration

### 3.1 Oversensitive perceptual input as the basis of weak central coherence, extreme systemizing, and communication deficiency

#### 3.1.1 Hyperacuity and overexcitability over sensory input likely start at early development

Widely observed cognitive underpinnings among ASC and other NDDs are the dominance of primary and local sensory information processing, the weakness of abstracting from that information, and having a global view. They also have difficulties combining information of different modalities over time. The peculiarities of vision perception have been extensively studied. This can lead to excellence in information processing in certain domains, such as in savants, and at the same time, difficulties in real-time responses needed for communication (201, 275). Perceptual distinctiveness observable daily is the *inflation or suppression of senses* in response to external stimuli (149, 276), a characteristic shared by the gifted (277). The high excitability of neurons because of insufficient suppression by GABA, and increased glutamine concentrations are considered physiological factors (201).

Brain development starts from lower-level sensory areas; subsequently, integration functions in the association areas and prefrontal lobes develop over a long period. The peculiarities of developmental trajectory in individuals with ASC are observable immediately after birth; their brain size is larger than that of TD individuals in the early stages of life (278), especially in the visual cortex and primary sensory areas, followed by the inferior temporal cortex (38) (**Figure 1**). Dominance of local visual processing seems to start in infancy (279), indicating that peculiarity in perception begins at the earliest development of primary sensory receptive fields, inducing the overdevelopment of certain perception-oriented information processing neural network traits, and neural excitability (280). Hypersensitivity of receptive fields and sensory processing in ASC individuals seems to be underlined by the peculiar density in neural cell packing; in ASC postmortem brains from infancy to young adults, mini-columns in the cerebral cortex were narrower in Brodmann area 3 (primary sensory area), 4 (primary motor area), 9 (prefrontal association area), 17 (V1), 21 (temporal visual association area), and 22 (temporal auditory area) (202, 281). In other specimens, ASC did not differ in cell structure in area V1 (Brodmann area 17), and narrowing of the mini-columns was most prominent in the ventral and orbitofrontal prefrontal lobes (282).

The function of estradiol on ERβ seems to be critical for early neural/glial distribution and closing the immature phase shifting to stimuli-dependent synapse modification (**Figure 2**). The time phases and pathways of neural maturation differ across perception modalities. In embryonic stem cell lines in which *SHANK3* was mutated into the ASC type, olfactory placodal neurons first emerged at the earliest stage of neurogenesis, and then developed smaller cell bodies and more and longer neurites compared to those in controls. These shifts were not observed in cortical neurons, except for the shortening of neurites (283). Similar changes were observed in the auditory areas, but in the opposite direction, with a wider interval between mini-columns compared to that in controls in the primary auditory and association fields. This difference was particularly significant in the primary auditory field in younger populations (284).

As stated previously, hyperacuity in the visual senses appears to be underlined by differences in neural cell structures. Notable characteristics of individuals with ASC include larger numbers of near-distance neural connections (285), a larger proportion of intra-hemispheric in contrast to inter-hemispheric neural connections, an inclination to excitation in the neural E/I balance, and heterogeneity of development between brain areas. The incoherence of processing and communication speed across modalities makes it difficult to combine information in real-time, resulting in the creation of unique information-processing networks that detour neurotypical processing in language areas, which may manifest as a unique brain torque (200) or non-right-handedness (286) in parts of the population.

#### 3.1.2 Visual imagery and synesthesia as the bases of savant ability

Typically appearing cognitive skills in savants are categorized as follows: 1) calendar calculation; 2) music; 3) art; 4) mathematical and number skills; 5) mechanical or spatial skills; and 6) “other obscure skills” which include exceptional multilingualism, sensory discrimination abilities, synesthesia, and knowledge in specific fields (121, 287). For ASC individuals, Temple Grandin grouped characteristic cognitive styles into the following: 1) visual thinkers; 2) music and math thinkers (pattern thinkers); and 3) verbal logic thinkers (288). Distinct cognitive strategies observable in high-functional ASC, savants, and gifted are calculation, architecture, and art creation by manipulating vivid visual imagery (68, 108–113). Enhanced pattern detection is likely to develop through exceptional sensory acuity and veridical mapping across isomorphic structures (289).

Individuals with ASC tend to preferentially use the visual cortex, in contrast to neurotypicals who use the prefrontal cortex to solve visuospatial tasks, such as mental rotation, under experimental conditions (93, 94). Furthermore, even in non-spatial tasks, such as the N-back working memory task which is typically processed verbally in neurotypicals, ASCs tend to use the right hemisphere and posterior cerebral regions, including the inferior temporal area and occipital lobe, indicating that they solve tasks visually (95). Inferior temporal gyrus comprises “what” ventral pathway in visual processing and responsible for *object imagery* handling color, texture, and patterns. The area stores single cells which index long-term memory, connecting semantic significance to the images, and also processes letters and manipulates numbers. Among individuals with ASC, visual imagery itself, focus on detail, and low communication ability were not related to savant abilities; however, high *spatial mental imagery* predicted a larger number of savant abilities (121). Spatial imagery is processed in the dorsal visual pathway leading to the occipito-parietal regions.

Synesthesia is another key to understanding savant phenomena through atypical sensory processing. In synesthesia, the senses of the different modalities are jointly perceived. Synesthesia is relatively common in ASC populations and closely associated with the emergence of savantism (290). Mental imagery ability is closely associated with sequence-space synesthesia, leading to savant abilities, such as calendar calculation (114, 115). The inferior temporal cortex and areas adjacent to the fusiform gyrus are responsible for grapheme-color synesthesia (291). In contrast, a survey of a larger population reported that the emergence of synesthesia was independent of the strength of visual imagery, and that individuals with weak visual imagery could possess synesthesia (292). Connectivity in the superior parietal or frontal cortex is responsible for the synesthesia irrespective of its subtype (291). Absolute pitch recognition is an aspect of veridical mapping and sound-related synesthesia that sometimes hinders language recognition, which requires the abstraction of information from similar phoneme patterns (289, 293).

In summary, the endophenotype connecting the cognitive characteristics commonly observed among ASC and other NDDs, savant and gifted individuals is heightened sensory sensitivity (121). The overdeveloped cortical parts in ASC high-risk infants overlap to those in male-to-female transgender individuals experiencing significant body dysphoria (transsexuals), in contrast to their cisgender counterparts in the postcentral gyrus (primary sensory area), left inferior temporal gyrus, and right lingual gyrus, respectively (38, 39). The other overdeveloped regions in the frontal and parietal occipital regions are located adjacent to each other (**Figure 1**). Lingual gyrus is responsible for visual imagery and creative thinking, in combination with other occipito-temporal-parietal complex areas (294). Another indicator that possibly reflects the early overdevelopment of sensory processing network is eidetic memory and memories from the early stages of life. Individuals with ASC have memories from an earlier age, not in an episodic form, but with sensory details (295).

#### 3.1.3 Untypical olfactory sensitivity

Olfactory sensitivity is another cognitive domain in which individuals with ASD do not simply exaggerate their TD male traits. Among TD individuals, olfactory sensitivity is superior in females, and some individuals with ASC individuals are obsessed with smell stimuli (68). Among the sensory symptoms, atypical sensitivity to taste and smell is the most prominent in ASC, and is a strong predictor of social deficits (72). Experimentally, some studies supported heightened sensitivity in trace smell perception threshold tasks in ASC (69), while others did not find differences from TDs (296, 297) or found blunter olfactory sensitivity (hypotonia) (73) and lower activity in the olfactory cortex (74), leaving the argument inconclusive (298).

In TDs, arousal in the autonomous nervous system is increased by the sniffing of human sweat during fear, but the response of ASCs was the opposite (299). The authors argued that social dysosmia (the inability to discriminate socially relevant chemosensory signals) is a causal factor in ASC’s incorrect reading of others’ emotions (298). Experiments using general odorant substances have shown that ASCs are rather impaired in the discrimination ability of odorants, and that the evaluation of smell stimuli is atypical (72, 75, 76).

In any standardized olfactory perception task to identify *sensitivity threshold*, *discrimination ability*, and *identification* with known stimuli, TD females outperform TD males, and the difference is most evident at *the threshold* (62), culminating in the ovulatory period when estrogen and androgen concentrations are the highest (67). In contrast, hyperosmia in women (strong sense of smell) is associated with morning sickness, but the emergence of morning sickness does not coincide with fluctuation of sex steroids, but with an increase in human chorionic gonadotropin (66). Pregnant women with morning sickness perceive the smell of food to be unpleasant. This does not necessarily correspond to a lowered threshold of olfactory senses; discrimination ability is undermined for some odorant stimuli (71). Unusual olfactory perception in ASC seems to be similar, reflecting a hyperreaction to perceived stimuli, but not necessarily the manifestation of heightened sensitivity (76).

Notable changes in the olfactory sensitivity have been observed in patients with epileptic seizures. In some cases, olfactory hypersensitivity and phantom odors are perceived as precursors or aftereffects of epilepsy (70). Epilepsy is found in 4–46% of individuals with ASC, and is also common in syndromes that contribute to ASC. An imbalance between excitatory and inhibitory neural activity is thought to be a common mechanism, and excessive estradiol activity in the brain during the critical period is a risk factor for epilepsy (192). Synesthesia sometimes emerges after epilepsy, drug intake, or brain trauma which cause the shuffling of neural sensory mapping in cortical layer V. Brain trauma converts neural environments into immature patterns, resulting in atypical neural circuitry in adults. Excitation of pyramidal neurons with the serotonin receptor, 5-HT_2A_, induces destabilization of the thalamus, possibly resulting in reinnervation with neural inputs from different modalities (300). Researchers have suggested similarities between hyperosmia, phantom odor, and synesthesia (301). Weakened inhibition from the frontal lobe due to a lesion in the right inferior parietal lobule is suggested as a trigger of hyperosmia (302).

### 3.2 Savants/gifted individuals excel in visual imagery processing, but are poor in spatial memory

#### 3.2.1 Cognitive and neurological profiles of individuals with SCA in reference to ASC

Brain morphology and cognitive characteristics in SCAs overlap with those in ASC. In studies on KS, the total cerebral volume was reduced, but the gray matter volume in the parieto-occipital and sensorimotor cortices were significantly increased (16, 303) or at least preserved (304), whereas the inferior frontal and temporal areas, especially in the dominant hemisphere, were reduced. A reduction in the frontal lobe corresponds to attenuated executive function, which is mostly evident in impaired mental flexibility (61). In the general male population with hypogonadism, there is evidence of an association between androgen deficiency and compromised executive function (151).

Although androgen therapy in adult KS is ineffective in improving mental flexibility, a mouse model suggested a combined negative effect of the number of X chromosomes and gonadectomy on the cognitive domain (77). Skews in cognitive domains (performance IQ>verbal IQ) overlap with other SCAs, except for Turner syndrome (karyotype XO) (53, 305). The ASC-like tendency was more severe in other SCA (XYY and XXYY) (58, 129) than in KS. Therefore, the general assumption is that the duplication of sex chromosomes, rather than sex steroid action mainly affects cognitive atypicality in SCAs (306). However, as suggested in Section 2.1.1, even XYY individuals which are generally categorized to possess typical steroid levels share some phenotypes suggestive of disruption of the steroid metabolism-feedback pathway, similar to other syndromes with hypogonadism. Chromosome-manipulated mouse models also suggest that the effect of chromosome number on cognitive differences is negligible, except when choosing same-sex conspecifics for mating, but is moderated by the organizational effect of sex steroids (307). A more comprehensive view is that the activational function of steroids is altered in individuals with SCA, as reported in animal models (308) and humans, probably through the alteration of organizational pathways. For example, the effect of CAG repeat on AR might be inverted in naturally hypogonadal populations, such as KS and female populations; in TD women, a longer CAG repeat, implying less active AR receptors, is associated with a higher circulating androgen concentration which is typically considered to be the marker of stronger masculinization (99).

Individuals with Turner syndrome are females with estrogen deficiency, characterized by superior verbal and poor visuospatial abilities (53). This pattern appears to be in contrast with KS and XYY, supporting the notion that sex chromosome allocation is crucial for cognitive deficiency and strength patterns. However, examination of task scores shows that they are more alike than the first look; impaired *visual memory* and *visual attention* are also observed in NDD with performance IQ strength, such as in individuals with ASC without superior IQ (82–84) or at older age (85). Visual memory corresponds to drawing complex figures through recall, using such as the Rey-Osterrieth complex figure test as a typical measuring tool. Androgen supplementation improves the scores in female-to-male transgender individuals experiencing significant body dysphoria (transsexuals) (78), and estradiol improves the scores in hypogonadal males (79), but is ineffective in women with Turner syndrome (86). In Turner syndrome, superior verbal ability is limited to certain domains, such as word knowledge, receptive/expressive abilities, verbal memory, coemerging with lower verbal fluency, articulation, and social skills. The prevalence rates of ASC, ADHD, and schizophrenia are greatly increased compared to those in TD women. Such overlaps of cognitive characteristics with KS and XYY suggest Turner syndrome’s similarity to ASC with hypergraphia (language production-strong subtype) (86).

A lack of optimal estrogen supply through critical periods throughout life leads to the suppression of verbal fluency, verbal memory, and executive function, and increases the risk of schizophrenia, ASC, ADHD, general anxiety disorder, and depression (222). Such cognitive profiles correspond to KS, XYY, and Turner syndromes. The general cognitive benefits of testosterone supplementation in KS (124) and general hypogonadal men (88, 125) are an increase in verbal fluency, but not in visuospatial ability. In a comparison between testosterone and DHT supplementation in hypogonadal men, only DHT was effective in improving spatial memory (route and spatial array learning tests), whereas testosterone improved verbal memory (story recall), suggesting that the latter effect occurs after conversion to estradiol (88).

Decreased brain volume in KS is known to occur in the hippocampus, insula, striatum, and amygdala (16). However, some studies did not find a reduction in the hippocampus (304). Spatial memories of complex figures and places can be attributed to the hippocampus. The hippocampus is abundant in both AR and ERβ (290), and estrogen deficiency impairs hippocampal development. In female mice, ERβ-KO causes a decrease in visuospatial learning ability (90). In KS, oral supplementation of the androgen, oxandrolone, in boys of 4–12 years was effective in increasing hippocampal volume to the TD range (80). The volume of the hippocampus positively correlated with scores on the Recall of Designs subtest of the Differential Ability Scale (81). Oxandrolone is a non-aromatizable androgen, but sometimes causes *gynecomastia* through an unknown loop. Sex steroid administration in gonadectomized male rats has revealed that the cognitive-enhancing effect of androgen on the hippocampus is at least partly through its action on ERβ (89).

A comprehensive review of the effect of estrogen on androgen-deprived males showed that estradiol treatment improved visual/verbal memory and behavioral inhibition (79). It should be noted that in the comparison of visuospatial tasks between animal models and humans, the latter depends on the manipulation of visual images, whereas the former depends on localization in actual spatial environments.

#### 3.2.2 Effect of steroid hormones across different components of visuospatial abilities

The EBM theory gives the impression that visuospatial abilities, such as mental rotation, are derived from higher androgen levels, and that female-favored verbal abilities are more facilitated by an increase in estrogen levels. Although previous reports have supported these ideas, recent findings have refuted such a simple relationship. The most robust sex differences in cognitive ability have been found in mental rotations using two-dimensionally depicted polygons (92). Lesbians and women with CAH exposed to high levels of androgens during fetal life have higher scores for mental rotation among women, whereas men with CAH have lower scores than typical men (96). Two explanations are possible for the latter association: androgen negative feedback may be in effect, resulting in lower androgen action and visuospatial ability, or the highly androgenic environment in the early male developmental period reduces the capacity for mental rotation. Animal models have also shown inconsistent results regarding the effects of CAH on males, and it cannot be determined which model is more appropriate (104). Additionally, in a meta-analysis of CAH studies, 2D:4D digit ratio was found to be an ineffective predictor of spatial ability (96).

A notable finding was that although boys with CAH were masculinized in hobby choice compared to their siblings, their spatial and mechanical abilities were lower (104), suggesting that higher prenatal androgen levels *compromised* their spatial and mechanical abilities. Boys with androgen excess disorders (Familial male precocious puberty) did not show an ability difference in a virtual water maze task compared to controls (309). A study of adult TD males found that mental rotation and spatial visualization scores (paper folding and MacQuarrie blocks) were negatively correlated with salivary T levels, whereas shorter AR CAG repeat polymorphisms (stronger androgen action) were associated with higher scores on spatial visualization tasks (97).

Visuospatial abilities, such as *mental rotation*, may be best in populations with testosterone levels intermediate between those of women and men (99, 100). A longitudinal investigation of cycle effects for eight weeks reported a quadratic association between salivary T and mental rotation scores within each sex (101). Among boys, gifted individuals had superior mental rotation ability, and higher scores were associated with lower prenatal androgen action, as suggested by longer AR CAG repeat lengths and larger 2D:4D ratios (215). Contradictory to this picture is a report that heterosexual and gay men without childhood gender nonconformity scored better in mental rotation than those with childhood gender nonconformity (102).

Although various reports have found no association between circulating androgen levels (103, 239, 310–312) or androgen administration and visuospatial ability, particularly among men (313), a robust relationship has been reported in which high circulating estrogen levels suppress mental rotational capacity. The mental rotation scores of naturally cycling women increase during periods of low estrogen concentration (314), correlating negatively with salivary estradiol levels (105). In men, lower estrogen concentrations were associated with higher mental rotation scores (103).

In contrast, s*patial memory*, which uses visuospatial working memory to remember where things are located, is better in women, and higher estrogen levels are more advantageous. Androgen-suppressed, TD men undergoing androgen deprivation therapy for prostate cancer, and long-term abusers of exogenous androgens have reduced visuospatial working memory (91), which can be caused by estrogen deficiency.

#### 3.2.3 Similarity of visual processing endophenotypes of dyslexia to ASC and savant/gifted in reference to KS

The most robust and prevalent cognitive profile of KS is a slightly inferior language ability, with *dyslexia* occurring in 50–70% of cases (52, 123); auditory processing and verbal memory are the weakest components (315). The cognitive characteristics of individuals with dyslexia have similarity with those of savants and gifted. A person may be gifted in mathematics, but has difficulty with sequential calculations and memorizing formulas (316). They also excel in various artistic activities and are superior at extracting patterns from visual noise (a measure of *object imagery*). Art students showed a higher rate of dyslexia (106, 122) and weaker speech perception skills.

In dyslexic brains, hypoactivation of the left occipital-temporal region during language tasks (317) and underdevelopment of the left frontal gyrus have been observed, especially among populations that use logographic letters, such as Chinese (318). Brains of KS have been reported to show a similar lack of dominance when processing language tasks (319, 320), although not all (321).

The total brain volume in KS is smaller than that in TD (322), with smaller volumes in the insula, frontal and temporal lobes, and subcortical regions, such as the amygdala, hippocampus (16, 51, 94, 95), and cerebellum (see Section 3.2.1). In contrast, the sensorimotor and parietal-occipital areas are enlarged, and the white matter volumes of the left cuneus and precuneus are the most robust, and extending to the inferior parietal regions in some cases (152, 303, 323). The inferior parietal lobule, which includes the angular gyrus (Brodmann area 39), is important for problem-solving through visual imagery, and is enlarged in mathematicians (107) and artists, especially in the non-dominant hemisphere (106). This region is responsible for connecting bodily sensations to spatial self-images. Stimulation of the angular gyrus induces out-of-body sensations, as is often observed in ASC, schizophrenia, and dissociative disorders (324).

It has been assumed that the blockage of language development and general intelligence might be complemented by other routes of information processing and outlets, as reported in the early cases of savants excelling in dancing or music (183). Well-known examples of *paradoxical functional facilitation* include acquired damage to the left hemisphere, resulting in savant-like abilities, including temporal lobe epilepsy, stroke in the dominant hemisphere, and frontotemporal dementia (287). It has been implied that damage to the frontal lobes enhances the function of the sensory areas in the posterior part of the brain (325).

A decrease in fine motor skills and visual-motor integration is also manifested in KS and XYY (53), and is ameliorated by androgen supplementation (142), which is probably also associated with improvements in verbal fluency. As observed in ASC, individuals with KS are weak at working memory (152), and they make more errors when presented with visual patterns that are difficult to discriminate, especially when presented sequentially (87). These phenotypes underlie the difficulties in language manipulation.

Autism spectrum conditions and predisposing syndromes of ASC often include a history of epilepsy (ASC: 4–46%, KS: 5–17%). Epileptic seizures alter neurotransmitter efficiency; however, only a few cases of acquired savants are directly attributable to epileptic seizures (325). Instead, endophenotypes that induce ASC, savant, or gifted individuals, in addition to syndromes with chromosomal abnormalities (326) and hypogonadism, should be considered to share a predisposition common to epilepsy. Atrophy of various brain regions has been observed in individuals with a long history of epilepsy (327). Epilepsy is caused by neuronal hyperexcitation, and astrocytic abnormalities are among its main factors. Sex steroid concentrations affect the severity and frequency of epileptic seizures. Notably, an overdose of estrogen aggravates neural overexcitation (328). Additionally, valproic acid, a major antiepileptic drug whose intake by the mother induces ASC in the fetus, disrupts the fetal HPG axis and causes autoimmune diseases in males (329).

### 3.3 High sensitivity to social cues, shyness, and impulsivity

#### 3.3.1 Shyness and high social anxiety, weaker interoception network, and alexithymia lead to insufficient communication skills

Contrary to the impression that individuals with autistic tendency are “mind-blind” and inattentive to social cues, the narrative of affected individuals support the view that they shut down the flow of social information, because they are afraid of getting panicked by emotional disturbance caused by involvement with others and their gazes (330). Individuals with KS (128) and at least subsets of ASC (331, 332) show increased physiological arousal in response to social cues, such as the human eye and mouth, but less activation of the amygdala and insula (333). It is difficult for them to assess and label affective responses, that underlies alexithymia (128). This makes them easily panicked by social stimuli; thus, they avoid them.

When individuals perceive their own emotions, information from the peripheral nerves inside the body, including visceral sensations such as heartbeat and temperature, are input via the insular cortex to the cingulate gyrus or the ventral medial frontal cortex. Conscious access to internal receptive sensation (interoception) is carried by the anterior insula and anterior cingulate gyrus, and is central to simulating the emotional states of others (143), thereby enabling to empathize with others.

Alexithymia does not necessarily mean that affected individuals lack an interoceptive sense or emotion; however, it makes it more difficult to explicitly identify emotions and communicate one’s feelings to others, thereby reducing the capacity to empathize with others (28, 143, 332). The insular cortex and cingulate gyrus are atrophied in KS (334), which corresponds to difficulty in social interaction and language development. Insular volume in ASC is not different from that in TD, whereas hypoactivation in emotional empathy task corresponds to higher levels of alexithymia (36). For KS (333) and ASC (36), the identification of stimuli with negative emotional valence is difficult or hypoactivates brain areas for processing emotions, which might also correspond to a reduction in amygdala volume. To complicate the interpretation, the insula in ASC shows hyperactivation in different types of tasks.

The most robust cognitive profiles in KS are shyness, unassertiveness, withdrawal, impulsiveness, and anxiousness (306). That is similar to that of ASC (335). Androgen replacement therapy for KS improves verbal IQ, and evidence suggests that this is largely due to reduced social anxiety (208). The reasoning is opposite to general human population, whose right anterior insula is overactivated in generalized anxiety disorders and in cases with high neuroticism, and these individuals show *high* interoceptive sensitivity (147).

In contrast to the fear of evolutionarily relevant stimuli, which is adaptive, anxiety is a high-vigilance response to undefined threats. Oxytocin acts to reduce anxiety in humans and animals, which helps to discriminate between actual danger and safety (336), and the candidate for its major activation site is bed nucleus stria terminalis (220, 337, 338). In TD participants, intranasal oxytocin administration during imaging social embarrassment situation experienced by self or others reduced physiological arousal and activation in the right amygdala and insula, especially among participants with high trait anxiety. This also increases the empathic ratings of others’ embarrassment and their own embarrassment, supporting the idea that attenuation of maladaptive vigilance to social stimuli attenuates alexithymia and helps empathy with others (144). Although it is debatable how circulating oxytocin levels correspond to its activity in the brain, those with high concentrations of salivary oxytocin experience stronger body ownership during the rubber hand illusion, suggesting that oxytocin facilitates to joint top-down model of self and interoceptive awareness of one’s own body (29, 30).

Oxytocin receptors exist in the insula, but their numbers are not large, and their expression corresponding to social behaviors is more apparent in the anterior cingulate cortex and amygdala (339, 340). With regard to control by sex steroids, although ERs are relatively sparse in the insula, estrogen directly facilitates descending sympathoexcitatory information transfer by affecting GABAergic neurotransmission (145). In the majority of oxytocin receptors in the brain, such as in the paraventricular nucleus (PVN), the receptors are co-expressed with ERβ and facilitated by estrogen action (220). Additionally, the dihydrotestosterone metabolite, 5 -androstane-3,17 -diol (3 -diol), also activates the oxytocin promoter through action on ERβ (221). This could explain the pathway by which androgen supplementation in KS reduces anxiety and improves verbal ability.

#### 3.3.2 Steroid deficiency decreases impulsive action, but increases impulsive choice

Impulsivity and aggression are generally considered greater in males and male prevalent NDD. Androgens are known to function in the limbic system to regulate reproductive function, aggressive behavior, and homeostasis. Specifically, AR is expressed in the brain reward system, which includes the mesocorticolimbic ventral tegmental area and nucleus accumbens, as well as the medial prefrontal cortex, in male and female rodents, non-human primates, and humans. Steroid hormone concentrations in the mesocorticolimbic system are more than double those in the circulatory system, and are still present six weeks after gonadectomy, indicating an important role for local androgen synthesis in the central nervous system (341). Androgens enhance perseverance toward activities for greater rewards and risk-oriented behaviors. Testosterone enhances tolerance to large uncertain rewards. In contrast, the predominant personalities of KS and ASC are shyness, social withdrawal, and clumsiness, and their impulsivity is the opposite of that of bold risk seekers.

Several theories have attempted to dissect the conceptualization of impulsivity. *Sensation seeking* by Zuckerman concerns the inclination toward reckless behavior to increase reproductive chances, and is higher in males (157), culminating in adolescence, and then decrease (154). The sensation-seeking behavioral list matches “masculine” risk-seeking behavior, and is suggested to be more strongly connected to reward processing than impulsivity (158). In contrast, *impulsivity* refers to a lack of self-control or deficiencies in response to inhibition. Impulsivity linearly decreases with age in the general population from approximately 10 years of age (154). Deficits in inhibitory executive function are commonly observed in patients with KS, ASC, ADHD, and gifted individuals. Typically developed males undergoing androgen *deprivation* therapy experience emotional lability and impulsivity (low inhibition) (156); a decrease in volume and neural activity in the prefrontal cortex likely underlies this effect (153).

In the experimental paradigm, impulsivity is measured separately using *impulsive action* and *impulsive choice* tasks (342). Both link to the dopamine system; impulsive action is assessed with stop signal and go/no-go tasks, which measure the reaction time that subjects must respond to ‘go’ stimuli, while inhibiting responses to occasional no-go/stop signals. In animal models, impulsive action is stronger in males (159) and impulsive action in females is positively correlated with circulating estradiol levels (160).

In contrast, *impulsive choice* concerns delay discounting, corresponding to a skew toward choosing an immediate small reward, in contrast to a delayed or uncertain larger reward. This is a model of ADHD and addiction, in which impulsive choices are stronger in females. This type of disinhibition is a model of impulsivity observed in syndromes with hypogonadism and male-prevalent NDD, and is attenuated by sex steroids. In laboratory animals, orchiectomy increases, and supraphysiological testosterone administration *decreases* impulsive choice in males (161). Although ovariectomy and estrous cycle do not affect the performance in female animals, women show a decrease of “now” bias in follicular phase, indicating dopamine signaling facilitated by estradiol rise, which speeds up subjective time perception and decrease impulsivity (164). In other words, the results suggest that estradiol deficiency affects frontoparietal circuits supporting working memory, and makes the subject feel that the passage of time is slower, combined with the effect on the hippocampus that is linked to discounting behavior, leading to impatient decision-making.

The developmental theory of ADHD also suggests that insufficient estradiol at the developmental stage leads to a hypofunctioning mesolimbic dopamine system and inefficient reinforcement, inducing the affected person to seek stronger stimulation (162). The authors suggested that suboptimal estrogen and dopamine activity decreases the opening time of NMDA receptors and hinders the development of association and behavioral learning.

## 4 Mismatch between top-down expectation and bottom-up perceptive input leads to unstable identity

### 4.1 Blockade of interoception leads to hallucination and diffusion of self-boundaries

The similarity between reports of unusual sensory/spiritual experiences and visual thinking of savant-gifted individuals was noted early in Gastaut–Geschwind syndrome (112, 116, 117). The inclination toward mysticism and excessive religiosity, which have often been seen in great individuals with special talents, are also characteristic of temporal/frontal lobe epilepsy, and some have suggested that these are features of ASC (68, 343–346). There is a category named the “hard-to-explain, paranormal sensations” among savant abilities (287), and gifted children show a tendency to have a precocious interest in self-transcendent questions and report hard-to-explain experiences (347).

As described in 3.3.1, KS and ASC show atypical activities of the insula and cingulate cortex, that joint internal perception (interoception) and the top-down concept of self, and constituting the Salience Network, which switches attention between executive function and internal mind-wandering. A decrease in activity in these regions has also been observed in individuals with schizophrenia, with an increase in the severity of hallucinations and heightened functional connectivity between the DMN and the Central Executive Network (37)(**Figure 1**). This implies that failure to switch to the executive network while attending to external stimuli constitutes the core mechanism that induces positive symptoms in schizophrenia (30, 348). In a typical conscious state, the projection pathway of interoceptive perception works independently of the neural pathways from the primary somatosensory cortex. Functional coupling between these factors using the hallucinogen, psilocybin, is a model of the unique perceptual experiences of savant-gifted individuals (118–120).

Bioactive compounds which work as psychedelics, such as serotonin, lysergic acid diethylamide (LSD), psilocybin, and dimethyltryptamine (DMT), act on the serotonin receptor, 5-HT_2A_, and mainly induce visual hallucinations and mystical feelings. Cortical hyperactivation of 5-HT_2A_ has been suggested to disrupt the thalamic gating of sensory and cognitive information (349, 350). Approximately one-third of the autistic population show 50–70% increase in blood serotonin levels (351, 352), which is called *hyperserotonemia*. Hyperserotonemia is more prevalent in the prepubescent and male populations. The density of serotonin transporters and 5-HT_2_ is decreased in adult ASC brains, especially in the anterior cingulate cortex (353, 354), suggesting the dysregulation of serotonin function. Selective serotonin reuptake inhibitors work on subsets of individuals with ASC to ameliorate adverse behaviors (352). Serotonin dysregulation is also observed in individuals with FXS (355). A delay in GABA shift disturbs the shift from serotonergic to dopaminergic neurons (**Figure 2**).

While individuals with schizophrenia experience depersonalization feeling and the sense of being manipulated by others, ASC has the sensory peculiarity as “difficulty in understanding the boundaries between self and others” (149). The central area that generates the subjective awareness of “self” as a reference to the surrounding environment is the cortical mediomedial structure within the DMN, which is active during self-resuming mind wandering (148). Activity in the DMN (medial prefrontal cortex, anterior/medial cingulate, and inferior parietal lobule), while performing self-referential tasks was consistently lower in the ASC in various experimental paradigms, suggesting that the highest-order self-concept is difficult to form (150).

The Bayesian brain model based on the free energy principle suggests that disruption of the interoception/Salience Network results in overfocusing on sensory input, and vice versa (28, 40, 41), which manifests as sensory oversensitivity in various NDDs and savants/gifted individuals (155). At the same time, the failure to select and hand over that external information to internal references, that form the concept of the world and self, results in a conscious state similar to that under the effect of sensory deprivation and hallucinogens.

## 5 Discussion and future direction

In this paper, we reviewed the evidence supporting that ASC is likely to co-occur with atypical sex differentiation, androgyny, contrary to the prediction by the EMB. We discussed its physiological factors and specificity of perceptual processing shared by savants and gifted individuals, with reference to the conditions of various sex differentiation diversities.

Although different neurosteroids affect the regulation of GABA shift (**Figure 2**) and subsequent behaviors in a unique manner (257), estradiol and ERβ seem to be among the main upstream factors that control early neural cell development and distribution, and the maturation timing of the developing brain. Theoretically, excess estrogen during early development can delay this shift; however, the observation of syndromic NDD cases and emergence patterns of giftedness suggest that hypogonadism is a more prevalent cause of such atypicality. Apparent hyperandrogenism can be explained by a reflection of deficiency in estrogen regulation in animal studies and human clinical cases. Combining the neurodevelopmental hypothesis with the psychological EPF model, we suggest a new paradigm to explain putative sex differences in strength/weakness in cognitive traits. They might be influenced by the balance of steroid actions, but not in a simple dichotomized manner, such as male-favor/female-favor. As it is indicated by strong neurosteroid function of estradiol, many apparent “male-favor” cognitive function is likely to be affected by estrogen or ERβ action (**Table 1**).

Variations in the endophenotypes of NDDs can be explained by alterations in shift timing and weight differences in each neural factor, caused by genetic and environmental influences. For example, the same SCA states show diverse manifestations of symptoms, ranging from none to ASC, schizophrenia, ADHD, and their combination. The insufficient functionality of androgen and estrogen is known to hinder the typical function of ASC-related genes, such as *FOXP2* and *SHANK3*, which can lead to an ASC-like phenotype. If only EPF is apparent, difficulties in language processing and symptoms of dyslexia may be more pronounced. If the imbalance between disinhibition of neural activity, control of executive function, and attention switching is more pronounced, it would lead to more ADHD-like features than ASC (356).

In humans, the critical period for the GABA shift extends postnatally to the first 12 months. The primary cause of shift delays and various NDDs is considered to be the downregulation of Potassium Chloride Cotransporter 2 (KCC2) (194). Steroid regulation of the shift varies across brain areas and with the subject’s sex, requiring a thorough examination of each endophenotype. Sex differences appear even before the peak of androgen secretion from the testes, suggesting a genetic background that affects endocrinological influences. Additionally, toxicological analysis has shown that the primary critical period for ASC is very early, starting from the time of neural tube closure to the end of second trimester. This occurs slightly later in schizophrenia, culminating between first and second trimesters (357). The risk periods extend to over three years of age for ADHD (357). The first and second trimesters are time windows for neurogenesis and neuronal migration (358). If estrogen deficiencies are critical in deriving NDD endophenotypes in hypogonadal individuals, as we suggested, the primary factor to produce ASC-like symptoms would be the insufficiency of ERβ functions in neural cell development, while each manifestation type will be later modified by the maturation shift in each neural system for individual timing.

The free energy principle suggests that in ASC, sensory perception is too precise, while top-down inference is weak, inducing a large Bayesian surprise at every time of perception, requiring recalculation (41). In contrast, perceptive information is less precise in schizophrenia, allowing inner models to go astray and elude from correcting inferences (359). Further studies are required to clarify when and how receptive precision diversifies across these two conditions.

The formation of an inner image and its extension to an atypical sensation, such as a feeling of dissociation or synesthesia, is a cue for understanding underlying mechanisms. Pharmacologically and epidemiologically, serotonergic hyperactivation of 5-HT_2A_ induces a mismatch between top-down self-image inference and bottom-up perception, transcending the boundaries of input source areas, leading to deep hallucinations, including ego dissolution (119). Thus, ASC and gifted individuals, not only those with schizophrenia, tend to have unusual beliefs. Serotonergic regulation is disrupted in ASC, while intracellular concentration of 5-HT is high in many of them. The extended immature phase before the GABA shift may be the cause of the atypicality of the serotonergic pathway; however, its interaction with steroids and oxytocin function is yet to be elucidated.

Recent progress in the therapeutic use of psychedelics is likely to open the way for answering this question. A drug called ‘ecstasy,’ 3,4-Methylenedioxymethamphetamine (MDMA) promotes affiliative feeling toward others. While MDMA does not bind to the 5-HT_2_ receptor, it induces the release of serotonin and oxytocin. This drug is effective in alleviating anxiety in individuals with ASC or post-traumatic stress disorder, opening a critical period for social reward learning neural circuits for reinnervation (360). A rodent study demonstrated that the critical period opening characteristic is not limited to MDMA, but is shared with LSD and psylocibin, which worked on 5-HT_2_ (361). Psychedelics with various activating targets result in the modulation of extracellular matrix, such as fibronectin, receptors, and proteases, allowing the re-wiring of the social reward learning system. The extent to which such after-corrections have a durable effect in individuals with altered endocrinological environments, such as hypogonadism, requires further investigation.

## Data availability statement

The original contributions presented in the study are included in the article/supplementary material. Further inquiries can be directed to the corresponding author/s.

## Ethics statement

Ethical approval was not required for the study involving humans in accordance with the local legislation and institutional requirements. Written informed consent to participate in this study was not required from the participants or the participants’ legal guardians/next of kin in accordance with the national legislation and the institutional requirements.

## Author contributions

KS: Writing – review & editing, Writing – original draft, Visualization, Investigation, Funding acquisition, Conceptualization. ST: Writing – review & editing, Investigation.
